# DCIS in BRCA1 and BRCA2 mutation carriers: prevalence, phenotype, and expression of oncodrivers C-MET and HER3

**DOI:** 10.1186/s12967-015-0698-3

**Published:** 2015-10-24

**Authors:** Rachel L. Yang, Rosemarie Mick, Kathreen Lee, Holly L. Graves, Katherine L. Nathanson, Susan M. Domchek, Rachel R. Kelz, Paul J. Zhang, Brian J. Czerniecki

**Affiliations:** Department of Surgery, Perelman School of Medicine, University of Pennsylvania, Philadelphia, PA USA; Department of Biostatistics and Epidemiology, Perelman School of Medicine, University of Pennsylvania, Philadelphia, PA USA; Department of Medicine, Perelman School of Medicine, University of Pennsylvania, Philadelphia, PA USA; Department of Pathology, Perelman School of Medicine, University of Pennsylvania, Philadelphia, PA USA; Abramson Cancer Center, 3rd Floor West 3400 Civic Center, Boulevard, Philadelphia, PA 19104 USA

**Keywords:** Breast cancer, Ductal carcinoma in situ, BRCA, Biomarkers, Prevention, Oncodrivers

## Abstract

**Background:**

Studies report conflicting evidence regarding the existence of a DCIS-associated premalignant pathway in BRCA mutation carriers. We aimed to examine the prevalence, phenotype, and expression of oncodrivers in pure DCIS (pDCIS) and invasive breast cancer with concurrent DCIS (IBC + DCIS) in mutation carriers.

**Methods:**

A cohort of BRCA1 and BRCA2 mutation carriers >18 years old who underwent surgery for breast cancer at an academic hospital (1992–2011) and had pathology available for review were included for study. Invasive breast cancer (IBC) and DCIS were stained for ER, PR, HER1, HER2, and HER3, and C-MET. DCIS prevalence was evaluated. Correlation of IBC and DCIS phenotypes was evaluated in patients with IBC + DCIS. DCIS and IBC expression of tumor markers were examined by BRCA mutation.

**Results:**

We identified 114 breast tumors. Of all BRCA1-associated tumors, 21.1 % were pDCIS and 63.4 % were IBC + DCIS. Of all BRCA2-associated tumors, 23.3 % were pDCIS and 60.5 % were IBC + DCIS. In BRCA1 and BRCA2 mutation carriers with IBC + DCIS, there was a significant correlation in ER, PR, and HER3 expression between the DCIS and IBC components. Most BRCA1-associated DCIS did not express ER, PR or HER2, while most BRCA2-associated DCIS did express ER and PR. BRCA1− as well as BRCA2-associated DCIS had expression of HER3 and C-MET.

**Conclusions:**

The majority of BRCA-associated tumors had DCIS present. Concordance of DCIS and IBC phenotypes was high, arguing for the existence of a DCIS-associated premalignant pathway. Oncodrivers HER3 and C-MET were expressed in the DCIS of mutation carriers, suggesting an opportunity for prevention strategies.

## Background

For individuals who carry a germline mutation in either the BRCA1 or BRCA2 gene, risk of developing breast and/or ovarian cancer is much greater than among the general population [1, 2]. However, mutation carriers only account for 7–10 % of breast cancers cases and 10–15 % of ovarian cancers cases [3–7]. Several studies have shown that the morphological and immunohistochemical phenotypes of BRCA1- and BRCA2-related breast cancers differ from that of sporadic breast cancers [8–10]. Compared to sporadic breast cancers, both BRCA1- and BRCA2-related breast cancers are more likely to be high grade and poorly differentiated [11–14]. It has been shown that BRCA1-related breast cancers have low expression of estrogen receptor (ER), progesterone receptor (PR) and human epidermal growth factor receptor (HER2/neu) as compared with sporadic breast cancers [15]. However, the ER and PR expression of BRCA2-related breast cancers does not seem to differ from that of sporadic cancers [16].

Much is known about the phenotypic differences between BRCA-associated and sporadic breast cancers, yet little is known about the differences in their pre-invasive progression pathways. Because more BRCA-related breast cancers are discovered between screening mammograms and with no prior pathologic findings [17, 18], it has previously been thought that the DCIS-associated premalignant pathway that exists among sporadic breast cancers is not present within the BRCA-associated disease spectrum. Studies have reported that DCIS is less often found near BRCA1- and BRCA2-associated invasive tumors when compared to sporadic tumors [19, 20]. However, more recent studies have found that high-risk pre-invasive lesions such as DCIS are more frequently found in prophylactic mastectomy specimens of BRCA mutation carriers than in control mammoplasty specimens [21–24].

Within the general population, DCIS has become a target for therapies aimed to prevent the development of invasive breast cancer. The relatively long period of latency between the onset of DCIS and development of sporadic invasive breast cancer has offered an opportunity to develop neoadjuvant interventions. Several neoadjuvant trials are underway for patients with DCIS, including anti-estrogen therapies and vaccines targeting HER2, both which have shown promise for patients with DCIS [25–27]. This hints at the possibility for new prevention options for patients with phenotypes underserved by currently available therapies, such as BRCA mutation carriers.

Today, the prevention of breast cancer among BRCA1 and BRCA2 mutation carriers has focused on surgical options such as risk-reducing bilateral mastectomy and bilateral salpingo-oophorectomy. Fortunately, these strategies have been shown to dramatically decrease the risk of breast cancer development [28, 29]. Nonetheless, there exist significant long-term consequences as well as effects on quality-of-life as a result of these surgical prevention strategies [30]. For this reason, efforts have been made to identify non-surgical prevention techniques for this patient population. Importantly, there is evidence that tamoxifen decreases risk of primary breast cancer as well as contralateral breast cancer for BRCA1 and BRCA2 mutation carriers [31, 32]. While studies are underway investigating additional options for chemoprevention in mutation carriers, there currently exist a paucity of non-surgical prevention strategies for this high-risk patient population.

Due to the substantial risk of breast cancer conferred by the BRCA1 and BRCA2 mutations, development of prevention strategies for mutation carriers is imperative. Chemoprevention strategies have been developed for the general population based on the known phenotypes of spontaneous DCIS and breast cancer, suggesting the importance of evaluating the phenotypes of hereditary breast tumors in order to develop targeted therapies. A growing body of literature supports targeting the HER family for prevention of ER-negative and possibly ER-positive breast tumors [33]. As such, investigating the phenotypes of BRCA-associated DCIS, specifically the HER tumor antigens, could elucidate possible targets to exploit in DCIS as a means of preventing invasive tumors in mutation carriers.

In this study we first aimed to identify the prevalence of pure DCIS and DCIS associated with invasive tumors among BRCA mutation carriers. We then aimed to assess the correlation between the phenotypes of invasive tumors and their corresponding DCIS in order to evaluate the role of DCIS in BRCA-associated tumor progression. Finally, we aimed to determine the unique immunophenotypes of BRCA1- and BRCA2-associated DCIS to investigate oncodriver expression that may be applicable to future prevention strategies.

## Methods

### Study population

**Fig. 1:**
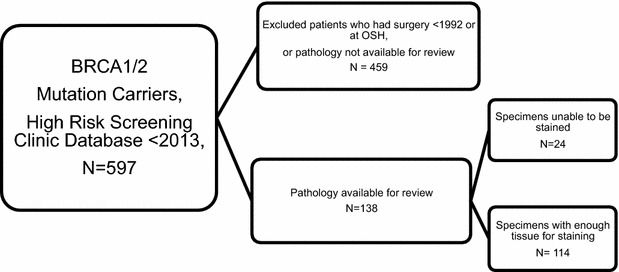
Criteria for study inclusion. BRCA1 and BRCA2 mutation carriers who were seen in the High Risk Screening Clinic before 2013 and were enlisted in the database for research were included for review. Patients who did not have pathology available, who were treated prior to 1992 (the year our electronic health record was introduced), or who treated at an outside hospital were excluded from the study. Patients who had tumor specimens without adequate tissue for staining were excluded from the study

After receiving approval from the Institutional Review Board at the University of Pennsylvania (protocol #814211), we obtained a list of patients seen in the High Risk Screening Clinic who were found to have a BRCA1 or BRCA2 mutation and were enlisted in the database for research purposes. We then restricted to mutation carriers who underwent a mastectomy or lumpectomy at our institution during the years of 1992–2011. We excluded all patients before 1992, because this was the year that the electronic pathology record system was first introduced at our institution. We then included only those patients who had pathology specimens available for review and who had specimens with sufficient tissue available for staining purposes (Fig. 1).

### Pathology review and staining

All breast cancer specimens were reviewed with a surgical pathologist for presence of DCIS and for tumor characteristics, including morphology of DCIS, distance of the DCIS from invasive tumor, and invasive and in situ nuclear grade.

All available pathology blocks for both invasive tumor and DCIS were cut and stained for estrogen receptor (ER), progesterone receptor (PR), human epidermal growth factor receptor 1 (HER1), human epidermal growth factor receptor 2 (HER2), human epidermal growth factor receptor 3 (HER3), and hepatocyte growth factor receptor (C-MET). In patients who had both invasive tumor and concurrent DCIS, we stained both the DCIS and the invasive components. All stains were interpreted by a single surgical pathologist. Both the percentage of positively stained nuclei and the intensity of staining (0–3) was recorded. An H-score was calculated for HER1, HER3, and C-MET by multiplying the percentage of positive nuclei by the stain intensity. Patients for whom one or more of the stains were unsuccessful or not interpretable were excluded from the study.

### Statistical methods

We examined patient characteristics by BRCA status using a Chi square test or *t* test, as appropriate. Associations between DCIS characteristics and DCIS prevalence, including pure DCIS and invasive breast cancer-associated DCIS, and mutation status were assessed by the Chi square test. In patients with invasive breast cancer with concurrent DCIS, Pearson correlation coefficients were calculated to determine correlation between HER1, HER2, and C-MET score in DCIS and invasive tumor, while a linear trend test was used to determine correlation between ER, PR, and HER2 intensity in DCIS and invasive tumor. Magnitude of DCIS and invasive tumor HER1, HER3 and C-MET score were compared by mutation status using the Student’s *t* test, while the Wilcoxon rank sum test was used to compare ER, PR and HER2 intensity.

Data management was performed using SAS Version 9.2 (SAS Institute Inc. 2009, Cary, NC, USA) and statistical analyses were performed using SPSS Version 21 (IBM Corp) or Stata/SE Version 11.1 (StataCorp, College Station, TX, USA). A p-value of <0.05 was considered significant for all statistical analyses.

## Results

We identified 114 breast tumors, of which 71 (62.3 %) were BRCA1-associated and 43 (37.7 %) were BRCA2-associated. Of all IBC, 80.2 % had concurrent DCIS. Of all BRCA1-associated tumors, 11 (15.5 %) were pure invasive tumors, 15 (21.1 %) were pure DCIS, and 45 (63.4 %) were invasive tumors with concurrent DCIS. Of all BRCA2-associated tumors, 7 (16.3 %) were pure invasive tumors, 10 (23.3 %) were pure DCIS, and 26 (60.5 %) were invasive tumors with concurrent DCIS. Prevalence of these three tumor types did not differ by mutation status (p = 0.95).

**Fig. 2:**
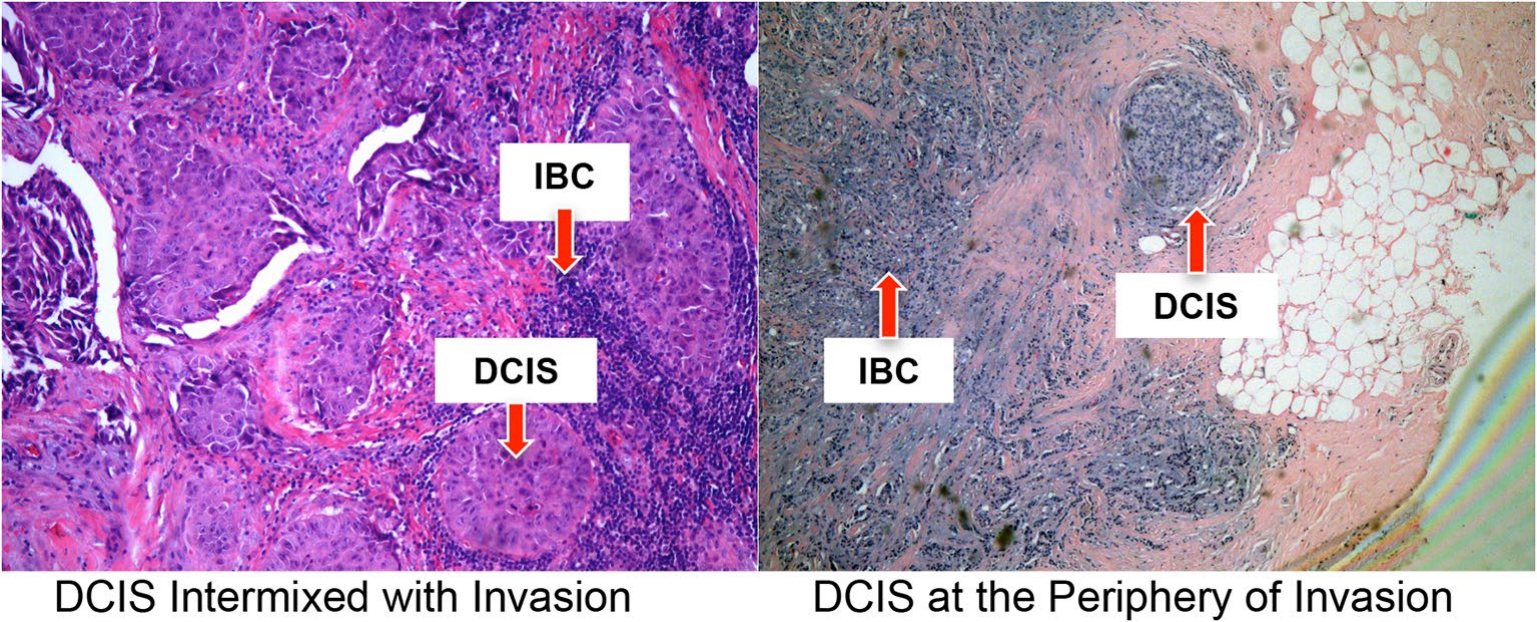
Appearance of tumors with both invasive and in situ components. The majority of DCIS was located on the periphery of the invasive tumor (<2 mm from invasion) or intermixed with it, not distant from the invasive tumor

When we examined the DCIS in tumors that had both invasive and in situ components, we found that the characteristics of the DCIS did not differ by mutation status (Table 1). For the majority of BRCA1- and BRCA2-associated tumors, the percentage of DCIS was less than 50 %, the DCIS morphology was comedo or cribriform, and the DCIS grade was high. For both BRCA1- and BRCA2-associated tumors, the majority of DCIS was intermixed with the invasive tumor or just on the periphery (<2 mm from the invasive tumor) (Fig. 2).

**Table 1:** Characteristics of DCIS found in BRCA mutation carriers with invasive tumors and concurrent DCIS

	BRCA1	BRCA2	P-value
	N (%)	N (%)	
% DCIS			0.14
<15 %	15 (53.6 %)	8 (32.0 %)	
15–49 %	9 (32.1 %)	8 (32.0 %)	
50+%	4 (14.3 %)	9 (36.0 %)	
DCIS morphology			0.10
Solid	4 (11.4 %)	3 (10.7 %)	
Cribriform	9 (25.7 %)	14 (50.0 %)	
Comedo	19 (52.3 %)	7 (25.0 %)	
Complex	3 (8.6 %)	4 (14.3 %)	
DCIS Distance from IBC			0.31
Intermixed	12 (42.9 %)	14 (56.0 %)	
Peripheral (<2 mm from IBC)	14 (50.0 %)	11 (44.0 %)	
Distant (>2 mm from IBC)	2 (7.1 %)	0 (0 %)	
DCIS Grade			0.63
Low	2 (5.7 %)	1 (3.6 %)	
Intermediate	11 (31.4 %)	12 (42.9 %)	
High	22 (68.9 %)	15 (53.6 %)

**Fig. 3:**
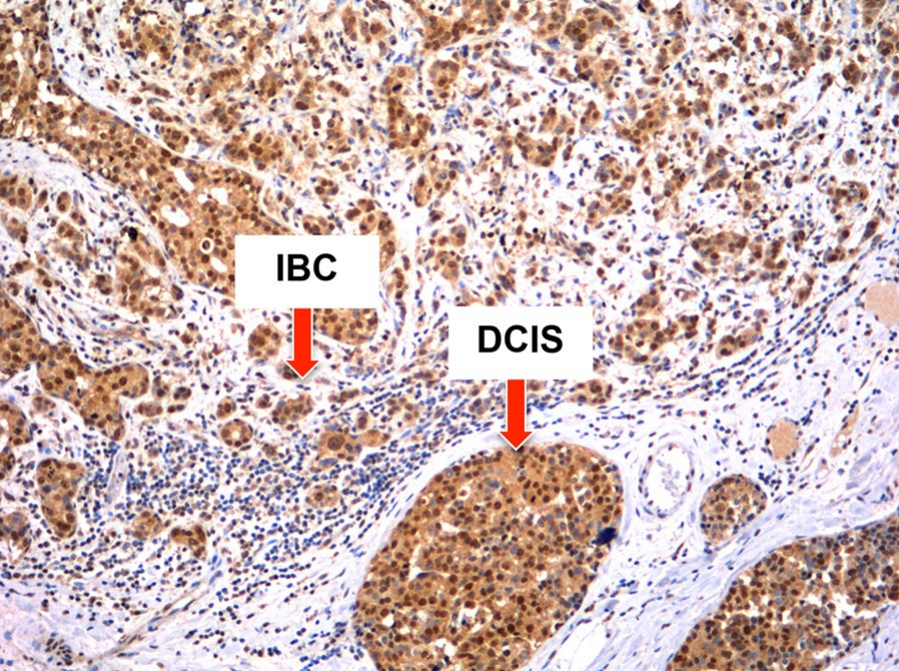
BRCA1 tumor with high correlation of DCIS expression of HER3 and adjacent invasive tumor expression of HER3

**Fig. 4:**
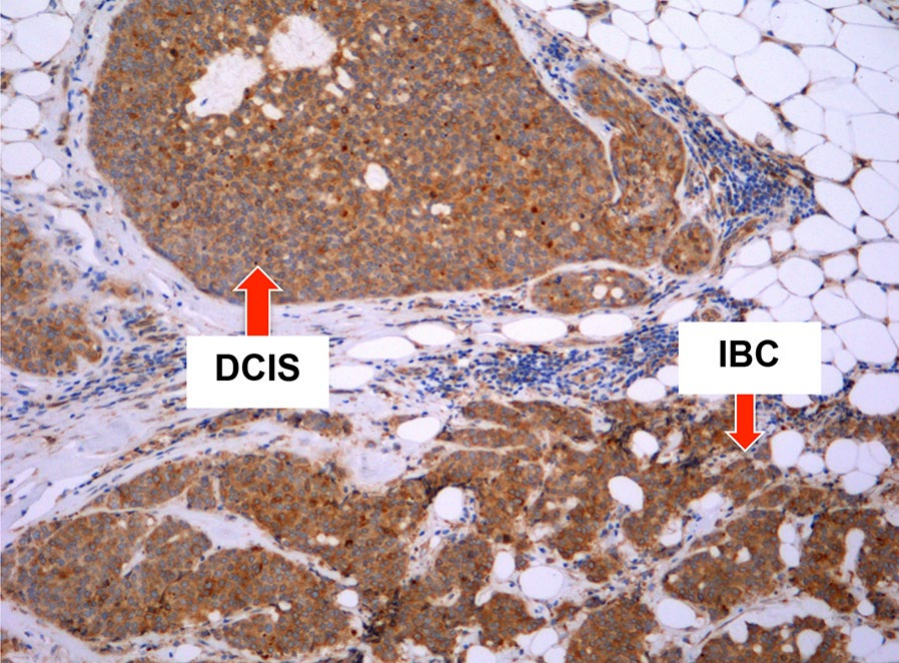
BRCA1 tumor with high correlation of DCIS expression of CMET and adjacent invasive tumor expression of CMET

When examining tumors that had both invasion and concurrent DCIS, we found the correlation between the invasive and in situ components to be high for most immunophenotypes. In BRCA1 mutation carriers with IBC + DCIS, the correlation between the DCIS and IBC (Tables 2, 3) was highly significant for ER, PR, HER1, HER3 (Fig. 3), and C-MET (Fig. 4). In BRCA2 mutation carriers with IBC + DCIS, the correlation between the DCIS and IBC was highly significant for ER, PR, HER2, and HER3.

**Table 2:** Correlation of IBC and DCIS expression of ER, PR, and HER2 in mutation carriers with IBC with concurrent DCIS, stratified by BRCA mutation

IBC ER intensity	BRCA1					BRCA2				
	DCIS ER intensity					DCIS ER intensity				
	0	1	2	3	P value*	0	1	2	3	P value*
0	23	0	2	1	<0.001	4	1	0	0	<0.001
1	1	0	0	0		0	0	0	0	
2	0	0	1	3		0	0	4	3	
3	0	0	0	2		0	0	1	8	

IBC PR intensity	DCIS PR intensity					DCIS PR intensity				
	0	1	2	3	P value*	0	1	2	3	P value*
0	23	0	3	2	<0.001	5	0	0	0	0.003
1	0	0	0	0		0	0	1	1	
2	0	0	0	1		0	0	2	2	
3	0	0	0	4		0	1	1	1	

IBC HER2 intensity	DCIS HER2 intensity					DCIS HER2 intensity				
	0	1	2	3	P value*	0	1	2	3	P value*
	16	5	0	0	0.46	7	5	0	0	<0.001
	6	4	0	0		1	6	0	0	
	2	1	0	0		0	1	0	0	
	0	0	0	0		0	0	0	2	

* Linear trend test for a table with ordered rows and columns

**Table 3:** Correlation of IBC and DCIS expression of HER1, HER3, and C-MET in mutation carriers with IBC with concurrent DCIS, stratified by BRCA mutation

	BRCA1			BRCA2		
	Pearson Correlation, r	N	P value	Pearson Correlation, r	N	P value
HER1 score	0.43	42	0.005	−0.09	23	0.69
HER3 score	0.87	25	<0.001	0.95	19	<0.001
C-MET score	0.47	29	0.01	0.34	17	0.19

Most BRCA1-associated DCIS and IBC had 0/3 staining intensity for ER, PR and HER2, while most BRCA2-associated DCIS and IBC had 3/3 staining intensity for ER and PR (Tables 4, 5). DCIS expression of ER, PR, and HER2 intensity was significantly higher in BRCA-2 tumors compared to BRCA-1 tumors (Table 4). IBC expression of ER and PR intensity were significantly higher in BRCA-2 tumors compared to BRCA-1 tumors (Table 5).

**Table 4:** Comparison of DCIS expression of ER, PR, and HER2 between BRCA1- and BRCA2-associated tumors

	BRCA1	BRCA2	Wilcoxon rank sum P value
ER intensity			<0.001
0	32	7	
1	1	1	
2	4	6	
3	7	14	
PR intensity			0.001
0	31	9	
1	2	1	
2	4	6	
3	7	13	
HER2 intensity			0.04
0	31	14	
1	14	14	
2	1	1	
3	0	2

**Table 5:** Comparison of IBC expression of ER, PR, and HER2 between BRCA1- and BRCA2-associated tumors

	BRCA1	BRCA2	Wilcoxon rank sum P value
ER intensity			<0.001
0	38	8	
1	1	0	
2	4	7	
3	2	11	
PR intensity			<0.001
0	40	10	
1	0	1	
2	1	5	
3	4	10	
HER2 intensity			0.56
0	33	19	
1	13	7	
2	3	2	
3	0	2	

BRCA1-associated DCIS had expression of HER3 and C-MET (H-Score 99.5 and 101.9, respectively), but lower expression of HER1 (H-Score 6.5), (Table 6). BRCA-2 associated DCIS also had expression of HER3 and C-MET (H-Score 84.3 and 124.8, respectively), but lower expression of HER1 (H-Score 16.5), (Table 6). DCIS expression of HER3, C-MET and HER1 did not differ significantly by mutation status. Similarly, BRCA1-associated IBC and BRCA2-associated IBC had expression of HER3 and C-MET, but lower expression of HER1 (Table 7), which did not differ significantly by mutation status.

**Table 6:** Comparison of DCIS expression of HER1, HER3, and C-MET between BRCA1- and BRCA2-associated tumors

	BRCA1		BRCA2		Student’s *t* test P value
	N	Mean ± SE	N	Mean ± SE	
HER1 score	56	6.43 ± 1.81	31	15.48 ± 10.43	0.40
HER3 score	38	99.47 ± 9.88	28	84.29 ± 11.60	0.32
C-MET score	39	101.92 ± 10.74	30	124.83 ± 9.31	0.12

**Table 7:** Comparison of IBC expression of HER1, HER3, and C-MET between BRCA1- and BRCA2-associated tumors

	BRCA1		BRCA2		Student’s *t* test
	N	Mean ± SE	N	Mean ± SE	P value
HER1 score	50	20.30 ± 5.59	29	11.38 ± 8.36	0.50
HER3 score	41	81.46 ± 7.84	22	72.95 ± 15.86	0.41
C-MET score	49	131.29 ± 9.35	21	131.67 ± 16.72	0.49

## Discussion

In sporadic breast cancers the molecular profile of DCIS and the genetic progression pathway from in situ to invasive cancer have been well characterized [34–40]. The aims of the current study were to determine the prevalence of DCIS, investigate the unique immunophenotypes of DCIS, and assess the relationship between the phenotypes of invasive tumors and their in situ counterparts, among BRCA mutation carriers. By means of this investigation we aimed to better understand BRCA-associated DCIS and its role in the hereditary tumor progression pathway.

We were able to analyze the pathology from 104 BRCA-associated breast tumors. As expected, only 23 tumors were pure in situ lesions, while the remainder were invasive tumors with or without concurrent DCIS. Over 80 % of all invasive tumors had concurrent DCIS. This is a relatively high rate of DCIS among BRCA-associated invasive tumors, compared with reports from prior studies that range from 20 to 56 % [41, 42]. Studies of sporadic invasive breast tumors have found rates of concurrent DCIS ranging from 56 to 71 % [20, 41]. Our study suggests that DCIS occurs within hereditary invasive tumors at a rate similar to that of sporadic tumors, further supporting the hypothesis that DCIS is a precursor to invasive carcinoma in BRCA mutation carriers. Additionally, our finding that most of the DCIS was high-grade suggests that the entrance point for BRCA-associated DCIS in the tumor progression pathway may be at the high-grade stage, unlike the progression pathway of sporadic breast tumors which is thought to begin with low-grade in situ disease [40, 43].

We found that among patients with invasive cancer with concurrent DCIS, the concordance of expression between the DCIS and invasive tumor was remarkably high for most biomarkers. Additionally, the majority of DCIS was found intermixed with the invasive tumor or in close proximity of it. These findings further support the existence of a DCIS-associated premalignant pathway among patients with BRCA mutations. Studies of sporadic breast tumors have similarly shown that the molecular profile and immunophenotype of DCIS usually parallels that of its invasive counterpart [38–40], supporting the popular believe that sporadic tumors progress through DCIS before developing into invasive tumors.

We found that most BRCA1-associated invasive and in situ tumors were triple negative, while most BRCA2-associated tumors expressed ER and PR but did not express HER2. Prior studies have similarly shown that BRCA1-associated tumors tend to be ER, PR and HER2 negative, while BRCA2-associated tumors are more often ER and PR positive [15, 16]. Our finding that ER, PR, and HER2 expression in mutation carriers mimics what has been demonstrated in many other studies, serves to validate and strengthen the other results of this current study. However, while most BRCA1-associated tumors were triple negative, still a substantial number of BRCA1-associated DCIS had expression of ER (20.8 %) or PR (20.8 %), which supports previous studies that have demonstrated the benefit of anti-estrogen therapy and oophorectomy in BRCA1 patients [28, 29, 32].

Few investigators have examined other oncodriver expression in DCIS of mutation carriers, and thus our study evaluating HER1, HER3, and C-MET adds to the current body of literature regarding immunophenotypes of hereditary breast cancer. We found that DCIS expressed HER3 and C-MET for both BRCA1 and BRCA2 mutation carriers. This finding begs the consideration of how to implement strategies to target these oncodriver signaling pathways in BRCA mutation carriers so to prevent DCIS and invasive tumors. As the current treatment options for mutation carriers with DCIS are quite limited, there is undoubtedly a need for the development of new techniques geared to halt the development of DCIS in this patient population. Given the results of our study, possible options for such an effort might include the development of vaccines to target HER3 and C-MET oncodriver signaling pathways, or the utilization of kinase inhibitors for prevention. Nonetheless, it is clear that BRCA mutation carriers are a unique patient population deserving further investigation, particularly in regards to prevention strategies that might one day be utilized to prevent the development of pre-invasive or invasive tumors.

There are several limitations to the present study. First, while our study was able to directly assess the prevalence and immunophenotypes of DCIS in patients with known BRCA mutations, our dataset did not include patients with sporadic breast tumors for comparison. As such, we could only speculate how the characteristics of DCIS among our patients might compare to that of non-mutation carriers examined in other studies. Secondly, women with BRCA mutations are a small patient population and thus the number of subjects available for study inclusion was quite limited. Furthermore, some of our patients were excluded from the study because their pathology was no longer available for review or the slides that were available did not have sufficient tissue for all stains to be completed. While we do not have any reason to believe that this group of excluded patients was inherently different from the group maintained for study inclusion, we cannot be certain that there was no skewing of our data as a result of those patients lost.

## Conclusions

In conclusion, we found that the majority of BRCA-associated tumors had DCIS present. Among tumors with both invasive and in situ components, the concordance of DCIS and IBC phenotypes was remarkably high, arguing for the existence of a DCIS-associated premalignant pathway. HER3 and C-MET were expressed in the DCIS of mutation carriers, suggesting an opportunity to target these oncodriver pathways as a means to prevent DCIS and invasive breast cancer. We hope future efforts will aim at investigating and implementing DCIS prevention strategies in mutation carriers.

## Abbreviations

ER: estrogen receptor
PR: progesterone receptor
HER1: human epidermal growth factor receptor 1
HER2: human epidermal growth factor receptor 2
HER3: human epidermal growth factor receptor 3
C-MET: hepatocyte growth factor receptor (C-MET)
DCIS: ductal carcinoma in situ
IBC: invasive breast cancer
pDCIS: pure ductal carcinoma in situ
IBC + DCIS: invasive breast cancer with concurrent ductal carcinoma in situ
